# Antioxidant and Antimicrobial Activity of Mexican Oregano (*Poliomintha longiflora*) Essential Oil, Hydrosol and Extracts from Waste Solid Residues

**DOI:** 10.3390/plants8010022

**Published:** 2019-01-17

**Authors:** Teresa Soledad Cid-Pérez, Raúl Ávila-Sosa, Carlos Enrique Ochoa-Velasco, Blanca Estela Rivera-Chavira, Guadalupe Virginia Nevárez-Moorillón

**Affiliations:** 1 Departamento de Bioquímica-Alimentos, Facultad de Ciencias Químicas, Benemérita Universidad Autónoma de Puebla, Edificio 105E, 14 Sur y Av. San Claudio, Ciudad Universitaria, Col. San Manuel, 72420 Puebla, Puebla, Mexico; teresolcid@gmail.com (T.S.C.-P.); raul.avila@correo.buap.mx (R.Á.-S.); carlosenriqueov@hotmail.com (C.E.O.-V.); 2 Facultad de Ciencias Químicas, Universidad Autónoma de Chihuahua, Circuito Universitario s/n Campus Universitario II, 31125 Chihuahua, Chihuahua, Mexico; bchavira@uach.mx

**Keywords:** *Poliomintha longiflora*, oregano essential oil, hydrosol, extracts from waste solid residues, antioxidant activity, antimicrobial activity

## Abstract

*Poliomintha longiflora* is a Mexican oregano, which has not been widely studied. This work aimed to describe the chemical composition, antimicrobial and antioxidant activities present in *P. longiflora* essential oil (EO), the hydrosol from EO extraction and extracts from waste solid residues (WSRs), identified as ethanol extract, ethyl acetate extract and the subfractions of ethanol and ethyl acetate extracts. The chemical characterization of the EO, hydrosol and WSR extracts was performed by GC–MS and HPLC. Their antioxidant activity was evaluated using two methods, and their antimicrobial activity was evaluated against *Escherichia coli*, *Staphylococcus aureus*, *Listeria monocytogenes*, *Bacillus cereus*, and *Salmonella* Typhimurium. Thirty-one chemical components were identified in the EO. The subfractions from the ethanol and ethyl acetate extracts contain methylmaleic anhydride, thymoquinone, thymol, carvacrol, thymol acetate, carvacrol acetate, and phenolic acids. The EO presented the highest biological activities for antioxidant (136.05 mg equivalent of ascorbic acid/g (AAE/g); IC50 83.70 μg/mL of 2, 2-diphenyl-1-picrylhydrazyl (DPPH)) and antimicrobial tests (minimal inhibitory concentration (MIC) value of 250–750 mg/L), while the hydrosol and the ethyl acetate extract from WSRs had the lowest antioxidant activity (14.16 and 12.29 mg AAE/g respectively), and the hydrosol had the lowest antimicrobial activity (MIC of 3000 mg/L). The data suggest that Mexican oregano *P. longiflora* hydrosol and extracts from waste solid residues can still have compounds with antimicrobial and antioxidant capacities.

## 1. Introduction

In recent years, the characterization of bioactive compounds from essential oils and plant extracts has been widely investigated for their use in the food and pharmaceutical industries [1,2]. Oregano is one of the aromatic plants used as a food additive to enhance the flavor of food. The most commonly commercialized oregano species are Greek oregano (*Origanum vulgare* ssp Hirtum (Link) Ietswaart) and Mexican oregano (*Lippia graveolens* Kunth or *Lippia berlandieri* Schauer) [3]. Mexico is the second largest oregano exporter [4], and Mexican oregano represents 35–40% of worldwide oregano production [5,6,7]. On the other hand, *Poliomintha longiflora* is one of the less commercialized Mexican oregano species (native from Coahuila and Nuevo León, Mexico) and it is commonly used as a substitute spice for European oregano [8].

Oregano essential oil (EO) quality depends on the thymol and carvacrol content; these isomers are responsible for oregano flavor [9,10] and their different biological properties include antioxidant [11], antimicrobial [12], acaricidal [13], antiparasitic [14], and antifungal [15] capacities. It is widely known that oregano EO is applied as a natural preservative, extending the shelf life of food products [16,17]. However, the application of oregano EO is limited due to its strong flavor.

During the extraction of EOs from plants and spices, a large number of by-products is produced, which are discarded although they can still contain a large number of bioactive compounds. As an example, the bioactive compounds from waste solid residues (WSRs) from *Salvia* sp. after EO extraction were obtained by solvent extraction aided with ultrasound [18]. The extraction of polyphenols from waste solid materials from the preparation of essential oils of *Lavandula intermedia* and *Thymus mastichina*, among other aromatic plants, demonstrated their antioxidant capacity [19]. Therefore, this study aimed to characterize Mexican oregano (*P. longiflora*) EO and its by-products and evaluate their antioxidant and antimicrobial activities.

## 2. Results and Discussion

### 2.1. Chemical Characterization of EO and Partial Chemical Characterization of Hydrosol and Extracts from Waste Solid Residues

The extraction yield of *P. longiflora* EO was 0.92 ± 0.03%, which is in the range reported previously, with yields of 0.7 and 1.67, respectively [20,21]. In general, the extraction yield of EO from oregano may vary from 0.1% to 3.0% depending on several factors including moisture content, plant type, morphology, and extraction conditions [22].

**Figure 1:**
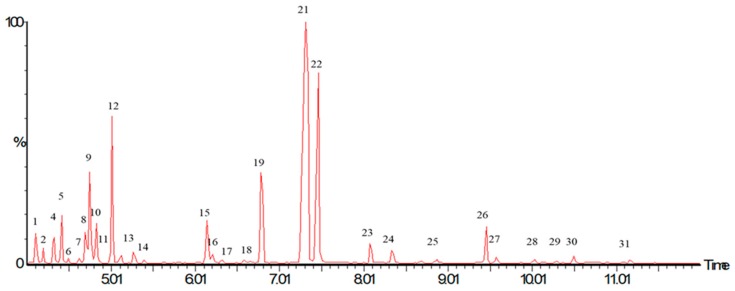
Chromatogram of *Poliomintha longiflora* essential oil obtained by hydrodistillation.

The analysis of the *P. longiflora* EO chromatogram (Figure 1) shows 31 different chemical compounds. Qualitative analysis showed that thymol, carvacrol, terpinolene, and carvacrol methyl ether are the major compounds of EO (Table 1). In 2009 [20], a total of 11 chemical compounds were reported in *P. longiflora* EO; all of them were also found in this study. Another report includes the concentration of the major components of *P. longiflora* (cultivated in 2010) and four compounds that were not reported before [8]. In a previous report, a difference in chemical composition was reported, with 36 and 31 chemical compounds in *P. longiflora* EO harvested in different years (2005 and 2006, respectively) [21]. A similar result was observed in 2016 for the EO of *O. vulgare* subs glandulosum, where the chemical composition depended on the harvest year, with 38, 33 and 28 compounds in 2007, 2008 and 2009, respectively [23]. Quantitative characterization of *P. longiflora* EO shows a higher concentration of thymol (1.97 ± 0.05 mg/mL) than carvacrol (0.89 ± 0.10 mg/mL). The results are comparable to those obtained by other reports, showing that thymol is the greatest compound in oregano Labiatae EO [24].

**Figure 2:**
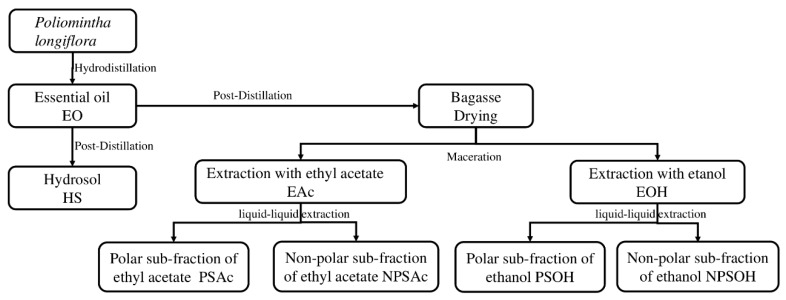
Flow diagram of *P. longiflora* essential oil (EO), hydrosol and extracts from the waste solid residues preparation procedure.

After EO extraction, the hydrosol or floral water was obtained (water remaining in the steam distillation equipment after EO extraction), and dried bagasse was also used for the preparation of ethanolic and ethyl acetate extracts, as identified in Figure 2. GC–MS analysis of the ethanol (EOH) and ethyl acetate (EAc) extracts was not possible due to interference in the samples, but in subfractions, six volatile components were characterized (Table 1). Thymoquinone, thymol, carvacrol, thymol acetate, and carvacrol acetate were not identified in the WSR ethanolic and ethyl acetate extracts by GC–MS analysis since their concentration was too low to be detected or it was not possible to separate them from noise. However, the compounds were concentrated in the subfractions, and it was possible to determine their relative abundance in the non-polar fractions.

In the polar subfraction of the ethyl acetate extract (PSAc) and the polar subfraction of the ethanol extract (PSOH), methylmaleic anhydride was detected; meanwhile, in the non-polar subfraction of the ethanol extract (NPSOH) and the non-polar subfraction of the ethyl acetate extract (NPSAc), thymoquinone, thymol, carvacrol, thymol acetate, and carvacrol acetate were detected. These last four compounds were also identified in the EO. A similar result was reported by Milos, which informed that thymoquinone, thymol, and carvacrol were determined in *O. vulgare* L. subs. Hirtum aqueous extract [25]. In this regard, it has been indicated that it is not possible to obtain the total extraction of volatile compounds from herbs and spices by hydrodistillation [26]; therefore, it is possible to extract volatile compounds from oregano bagasse using different solvents.

Caffeic and rosmarinic acids (non-volatile compounds) were determined in extracts by HPLC. All extracts obtained from waste solid residues of oregano after EO extraction presented rosmarinic acid (Table 1); however, caffeic acid was found only in residual water and polar subfractions. Regarding extracts from waste solid residues from the extraction process in *P. longiflora*, it was not possible to detect caffeic acid, but it was detected in the polar subfractions of the WSR solvent extracts. The concentration achieved during subfraction preparation led to the detection of caffeic acid in these samples, while only trace amounts that were undetected by the analytical method may have been present in the extracts. This information is consistent with results described by various authors who determined that the main phenolic acid of the Lamiaceae family is rosmarinic acid [27,28,29], while caffeic acid was found in minimal concentrations in *O. onites* L. extracts [28]. High concentrations of rosmarinic acid and trace concentrations of caffeic acid in ethanolic extracts of *O. vulgare* have also been reported before [30].

### 2.2. Total Phenols and Antioxidant Activity

The quantification of total phenolic compounds in the hydrosol and WSR extracts from the hydrodistillation of *P. longiflora* has not been reported before. Table 2 shows the total phenol (TP) quantification and antioxidant activity of *P. longiflora* Mexican EO oil and its by-products. As expected, the higher content of TPs was obtained in EO, followed by EOH and EAc. The results obtained in this study for EO (27.85 mg equivalent of gallic acid/g (GAE/g)) were higher than those reported in 2013 for *O. vulgare* EO (16.30 mg GAE/g) [31]. On the other hand, higher concentrations of phenolic compounds (35.40–55.40 mg GAE/g) have been reported in the clonal and commercial oregano ethanolic extracts, respectively [32]. As observed, the TPs obtained in each extract were significantly affected (*p* < 0.05) by the solvent used; in this aspect, EOH showed the highest TPs, while EAc presented the lowest concentration of TPs.

The antioxidant activity, expressed as the iron reduction of ferric ion, indicated that EO is the best antioxidant agent with 136.07 mg of AAE/g, followed by EOH (26.10 mg equivalent of ascorbic acid (AAE/g)) extract. Teixeira reported a lower reducing power of *O. vulgare* EO (74.5 μmol AAE/g) [31] than what we present here. On the other hand, the EAc and non-polar extracts presented a similar reducing power activity, while the polar subfractions had the lowest antioxidant values for this test.

The determination of 50% inhibition (IC50) in 2, 2-diphenyl-1-picrylhydrazyl (DPPH) scavenging activity is usually done to compare the antioxidant capacity of different antioxidant compounds. Similar to the TPs and ferric ion reducing test, the EO showed the highest antioxidant capacity (83.70 ± 4.12 μg/mL DPPH). In this regard, the values obtained in this study are lower (higher antioxidant activity) than those reported in 2004 and 2010 for the EO of *O. vulgare* sbsp Hirtum (500 µg/mL DPPH) [33] and *O. onites* L. (116.74–132.93 µg/mL DPPH) [28], respectively. This variation may be attributed to the difference in the chemical composition of oregano. Moreover, the aqueous and bagasse extracts still had antioxidant capacity due to the rosmarinic and caffeic acid (structure 4 hydroxyl and 2 hydroxyls groups, respectively) and trace compounds of EO. Although the aqueous extract and bagasse are considered residues, in *P. longiflora*, these by-products present similar IC50 values to those reported in a methanolic extract of *L. gravelolens* (152–207 μg/mL DPPH) [34] and in *O. vulgare* L. aqueous extract (335.0 μg/mL DPPH) [35].

### 2.3. Antimicrobial Activity

Different authors have concluded that the antimicrobial activity of oregano EO is attributed to its hydrophobic phenolic compounds [36,37,38], which interact with phospholipids that are present in the cell membrane. The results obtained (Table 3) indicated that *P. longiflora* EO showed better biological activity against Gram-positive bacteria than Gram-negative bacteria. Similar results were obtained by different researchers for *O. vulgare* EO [39,40,41,42,43,44]. The higher resistance of Gram-negative bacteria to EO is attributed, in part, to the complexity of the double cell membrane present, in contrast to the structure of Gram-positive bacteria [45].

Hydrosol had a mild antimicrobial activity and was more effective against *S. aureus*. Although the volume needed for antimicrobial activity against most of the tested microorganisms can be considered high (3000 mg/L or higher), the hydrosol was not concentrated, and the bioactive compounds present can, therefore, be found in low concentrations. Regarding the ethanolic and ethyl acetate extracts, the minimal inhibitory concentration (MIC) values were high as compared with the EO, but antimicrobial activity can still be considered important for *S. aureus* and *B. cereus*.

The non-polar subfractions contained a small number of volatile components and a high chlorophyll concentration, and the green color of the solution interfered with MIC determination; therefore, only the minimal bactericidal concentrations (MBCs) are reported (Table 3). From the subfractions, the PSAc had MIC values of 500–750 mg/L, while the NPSAc had a bactericidal activity of 750 mg/L against S. aureus, and 1000 mg/L for all the other strains tested. On the other hand, *B. cereus* was the most sensitive bacteria, followed by *S. aureus* and *E. coli*. Based on the data of MBC, the hydrosol, the ethanolic and ethyl acetate extract as well as the subfractions from the ethanolic extract, did not present bactericidal activity against *L. monocytogenes* and *S.* Typhimurium at the tested concentration. The antimicrobial activity of the extracts and subfractions can also be attributed to phenolic acids present in the samples, but not determined in this research.

## 3. Materials and Methods

### 3.1. Plant Material

The leaves and stems of *P. longiflora* were provided by the Cirena (Centro de Investigación en Recursos Naturales, Salaices López Chihuahua, Mexico). The *P. longiflora* was authenticated by botanists of the Department of Biology of Benemérita Universidad Autónoma de Puebla, Puebla, Mexico by visual inspection and comparison with the herbarium reference by a database. The plant material was used to obtain the essential oil (EO), aqueous extract and bagasse.

### 3.2. Essential Oil Extraction

Four hundred grams of dry powder of *P. longiflora* was placed in a Clevenger-type apparatus for 3 h. The EO was dried using anhydrous sodium sulfate and stored at 4 °C until used. The EO yield was obtained as the relation between the weight of dry EO and dry matter of *P. longiflora* Mexican oregano.

### 3.3. Preparation of Hydrosol and Waste Solid Residue Solvent Extracts

Hydrosol (HS) was considered as the water retained in the distillation flask at the end of the oregano hydrodistillation process. The residual water was filtered (Whatman grade 4) two times and kept in the dark at 4 °C in liquid form for further analysis. Oregano bagasse was considered as the waste solid residues left after the distillation for EO preparation. The bagasse was used to obtain both ethanol extract (EOH) and ethyl acetate extract (EAc) by maceration, according to the methodology proposed in 2003 [30]. The waste solid residues (free of EO) were filtered from the hydrosol and dried at room temperature for 24–48 h. The dried bagasse (18 g) was placed in an amber flask, 160 mL of either ethanol or ethyl acetate (J.T. Baker, Mexico City, Mexico) was added, and the flask was hermetically closed. The flask was left undisturbed for 24 h in a dry, dark place, and was further filtered (Whatman grade 4) and concentrated in a rotary evaporator (total dryness) for its analysis. The WSR extracts, EOH and EAc, showed a green coloration due to high chlorophyll content (yield of 16.93 and 35.98 mg/g of oregano WSR, respectively).

The ethanol and ethyl acetate bagasse extracts were further separated by liquid–liquid extraction. To an aliquot of 50 mL of the ethanol or ethyl acetate extract, without concentration, a formic acid solution (1% *v/v*) was added to achieve a final pH value of 2. The mixture was placed in a separation funnel and chloroform was added for the liquid–liquid extraction procedure. The formic acid-soluble fraction was the polar phase and was transparent, i.e., chlorophyll-free; the organic non-polar phase chloroform-soluble contained an elevated concentration of chlorophyll and was green. The subfractions were identified as a polar subfraction of ethanol extract (PSOH), a polar subfraction of ethyl acetate extract (PSAc), a non-polar subfraction of ethanol extract (NPSOH) and a non-polar subfraction of ethyl acetate extract (NPSAc). The yields of the ethanolic subfractions, PSOH and NPSOH, were 19.91 and 25.13 mg/g of extract respectively, and the yield of the ethyl acetate subfractions was 12.76 mg/g of PSAc and 40.5 mg/g of NPSAc. The polar and non-polar extracts were concentrated until total dryness. Figure 2 illustrates the preparation of all extracts from *P. longiflora*.

### 3.4. Partial Chemical Characterization of EO, Hydrosol, and WSR Extracts

Mexican oregano EO, the hydrosol and the extracts and subfractions obtained from oregano bagasse were analyzed using a GC Perkin Elmer Turbo Mass Gold MS-Auto system XLTM (Perkin-Elmer, Norwalk, CT, USA) with a splitless injector and 70 eV electronic fragmentation detector, equipped with an AT-1 capillary column (30 m × 0.25 I.D. × 0.25 μm). Helium was used as the carrier gas, and the following conditions were set for the analysis: the temperature of the injector and detector was 220 °C; the initial oven temperature was 120 °C held for 1 min, followed by a ramp-up of 3 °C/min up to 180 °C, and a second ramp-up of 25 °C/min up to 225 °C, and held at the final temperature for 7 min, with a flow of 10 mL/min. For all samples (EO and extracts, at an initial concentration of 10 mg/mL), ethyl alcohol was used as solvent for GC–MS analysis; the volume used for injection was 1 µL, and the relative abundance was determined for each of the components identified in the chromatogram. The spectra obtained were compared with the mass spectra for the respective pure compounds and with the mass profile of the same compounds available from the US National Institute of Standard Technology (NIST) library. For the MS analysis, the molecular weight range was of 35–430 *m/z*. The chromatographic peaks were identified by comparison with their Kovats retention indices (RI), based on mass spectral-retention index libraries, considering the temperature of analysis [46]. Thymol and carvacrol were further quantified by external standard calibration (0.5 to 5.0 mg/mL), using the pure compounds (Sigma-Aldrich, St. Louis, MO, USA). In all extracts, the rosmarinic and caffeic acids were elucidated using HPLC (Agilent model 1000, Palo Alto, CA, USA) with a zorbax eclipse C18 reverse phase column (5 μm in particle size, 250 mm in length and 4.6 mm in inner diameter) at 30 °C, with a wavelength of 280–520 nm, and a flow rate of 1 mL/min, using the following solvent gradients: gradient A—90% water-ATF 0.1%; and gradient B—10% ethanol.

### 3.5. Determination of Total Phenolic Content

The quantification of total phenols (TPs) was done according to previous work with some modifications [47]. For the extracts, ethanol was used as solvent. In an amber glass, (2.23 mL) distilled water was mixed with (150 µL) Folin–Ciocalteau reagent (Sigma-Aldrich, St. Louis, MO, USA) and (20 µL) the EO or extract. The mixture was left to stand for 10 min at room temperature. Afterwards, (600 µL) the Na_2_CO_3_ (15% *w/v*) (Sigma-Aldrich, St Louis, MO, USA) was added. The mixture was stirred and incubated at 40 °C for 20 min. The TPs were determined using a UV–Vis spectrophotometer (Perkin Elmer, Lambda 25, Waltham, MA, USA) at 760 nm. The TPs were calculated as mg equivalent of gallic acid (GAE) per g of EO or g of dry weight in extracts, using a standard curve (slope = 0.0211 mL/mg GAE; intercept = 0.0155 abs; R^2^ = 0.993) of gallic acid (Sigma-Aldrich, St. Louis, MO, USA). For the EO and extracts, an initial concentration of 10 mg/mL diluted in alcohol was used.

### 3.6. Antioxidant Activity

The iron reduction assay was determined according to the technique previously reported [48]. For the extracts, ethanol was used as solvent. The EO or ethanolic extract (1 mL) was mixed with (2.5 mL) phosphate buffer and (2.5 mL) a solution of K_3_[Fe(CN)_6_] (1%) in an amber glass tube. The mixture was incubated in a water bath at 50 °C for 30 min, then (2.5 mL) trichloroacetic acid (J.T. Baker, Mexico City, Mexico) was added to it before it was centrifuged (International Equipment, 4279M-6, Nashville, TN, USA) for 20 min at 1800 rpm. An aliquot (2.5 mL) of the supernatant was taken and mixed with (2.5 mL) distilled water and (0.5 mL) FeCl3. The absorbance was determined using a UV–Vis spectrophotometer at 700 nm (Perkin Elmer, Lambda 25, Waltham, MA, USA). Ascorbic acid (J.T. Baker, Mexico City, Mexico) was used as the standard (slope = 8.66 mL/mg AAE; intercept = 0.017 abs; R^2^ = 0.991). The results were expressed as mg equivalent of ascorbic acid (AAE) per g of dry weight. For the EO and extracts, an initial concentration of 10 mg/mL diluted in alcohol was used.

The free radical scavenging capacity was evaluated using the DPPH (2, 2-diphenyl-1-picrylhydrazyl) radical. In an amber glass tube, an aliquot (50 µL) of EO or the extracts at different concentrations (0.000, 0.025, 0.125, 0.250, 0.375, and 0.500 M) was added with (1.95 mL) DPPH (Sigma-Aldrich, St. Louis, MO, USA) methanolic solution (9 × 10^−5^ M). The mixture was stored in a dark environment at room temperature until the reaction was stable (1 h approximately). The absorbance was determined using a UV–Vis spectrophotometer at 517 nm (Perkin Elmer, Lambda 25, Waltham, MA, USA). The results were expressed as IC50 (mg/mL DPPH) values.

### 3.7. In Vitro Antimicrobial Test

The *P. longiflora* EO and extracts were evaluated against *Salmonella* Typhimurium ATCC 14028, *Staphylococcus aureus* ATCC 25923, *Listeria monocytogenes*, *Bacillus cereus* ATCC 11778 and *Escherichia coli* O:157H7 ATCC 43888 following the methodology proposed by Hammer et al. [49]. The microorganisms were grown to exponential phase (1.5 × 10^8^ CFU/mL) in nutrient broth (BIOXON, Mexico city, Mexico) at 35 °C for 18 h. One milliliter of the inoculum was transferred to 9 mL of trypticase soy broth, and the EO or extracts at different concentrations (50, 100, 250, 500, 750 and 1000 mg/L) were added. The samples were incubated at 37 °C for 24 h. Minimal inhibitory concentration (MIC) values were determined as the lowest concentration without observed growth. Inoculated tubes without EO or extracts were used as the control. For the determination of the minimal bactericidal concentration (MBC), a loopful of tubes with no visible growth after incubation was inoculated in Trypticase Soy Agar and incubated at 37 °C for 24 h. The MBC was reported as the concentration without microbial growth detected in plates.

### 3.8. Statistical Analysis

The experiments were done in triplicate. The results were statistically analyzed by ANOVA using the Minitab 14 program (Minitab Inc., State College, PA, USA). A *p*-value of 0.05 was used to determine significant differences among averages with Tukey’s test.

## 4. Conclusions

The chemical characterization of *P. longiflora* Mexican oregano essential oil has shown thymol and carvacrol as the major components, along with other volatile compounds to sum a total of 31 identified components. The partial chemical characterization of the hydrosol, as well as the solvent extracts and subfractions obtained from waste solid residues, demonstrated the presence of caffeic acid, rosmarinic acid, methylmaleic anhydride, thymoquinone thymol, carvacrol, thymol acetate, and carvacrol acetate; these last four compounds were also identified in the EO. *P. longiflora* EO and the extracts presented an antioxidant capacity and a mild antimicrobial activity. Therefore, the hydrosol and the waste solid residue or bagasse left after EO extraction can be further used as a source of bioactive molecules.

## Figures and Tables

**Table 1:** Chemical composition, retention index, and retention time (min) of *P. longiflora* Mexican oregano essential oil, hydrosol and extracts from waste solid residues.

Peak	Component	RI	RT (min)	EO% *^c^*	HS% *^d^*	EOH% *^e^*	EAc% *^f^*	PSOH% *^g^*	PSAc% *^h^*	NPSOH% *^i^*	NPSAc% *^j^*
1	Thujene *^a^*	924	4.11	2.41	---	---	---	---	---	---	---
2	a-Pinene *^a^*	932	4.2	1.66	---	---	---	---	---	---	---
3	Methylmaleic anhydride *^a^*	949	4.39	ND	---	---	---	2.17	9.45	---	---
4	Octen-3-ol *^a^*	974	4.31	2.24	---	---	---	---	---	---	---
5	b-Pinene *^a^*	974	4.43	3.57	---	---	---	---	---	---	---
6	Myrcene *^a^*	988	4.53	0.3	---	---	---	---	---	---	---
7	Phellandrene *^a^*	1002	4.63	0.25	---	---	---	---	---	---	---
8	4-Carene *^a^*	1008	4.7	2.76	---	---	---	---	---	---	---
9	p-Cymene *^a^*	1020	4.76	6.7	---	---	---	---	---	---	---
10	Limonene *^a^*	1024	4.83	2.81	---	---	---	---	---	---	---
11	Ocimene *^a^*	1032	4.87	0.02	---	---	---	---	---	---	---
12	Terpinolene *^a^*	1086	5.02	6.96	---	---	---	---	---	---	---
13	Linalool *^a^*	1095	5.28	1.12	---	---	---	---	---	---	---
14	Sabinene hydrate *^a^*	1098	5.41	1.33	---	---	---	---	---	---	---
15	Borneol *^a^*	1165	6.14	4.36	---	---	---	---	---	---	---
16	Terpinen-4-ol *^a^*	1174	6.22	0.24	---	---	---	---	---	---	---
17	a-Terpineol *^a^*	1186	6.33	0.25	---	---	---	---	---	---	---
18	Thymol, methyl ether *^a^*	1232	6.58	0.22	---	---	---	---	---	---	---
19	Carvacrol, methyl ether *^a^*	1241	6.66	7.81	---	---	---	---	---	---	---
20	Thymoquinone *^a^*	1252	7.19	ND	---	---	---	---	---	2.25	3.04
21	Thymol *^a^*	1289	7.33	28.31	---	---	---	---	---	35.44	42.12
22	Carvacrol *^a^*	1298	7.48	17.06	---	---	---	---	---	20.75	30.08
23	Thymol acetate *^a^*	1349	8.09	0.84	---	---	---	---	---	2.92	3.62
24	Carvacrol acetate *^a^*	1370	8.36	0.71	---	---	---	---	---	3.82	4.12
25	Bourbonene *^a^*	1387	8.88	0.10	---	---	---	---	---	---	---
26	b-Caryophyllene *^a^*	1408	9.47	1.31	---	---	---	---	---	---	---
27	Farnesene *^a^*	1440	9.58	0.08	---	---	---	---	---	---	---
28	Muurolene *^a^*	1478	10.04	0.08	---	---	---	---	---	---	---
29	Germacrene D *^a^*	1484	10.3	0.08	---	---	---	---	---	---	---
30	Germacrene A *^a^*	1508	10.51	0.1	---	---	---	---	---	---	---
31	a-Cadiene *^a^*	1537	11.17	0.09	---	---	---	---	---	---	---
32	Caffeic acid *^b^*	---	20.92	---	1.56	---	---	1.85	1.35	---	---
33	Rosmarinic acid *^b^*	---	37.98	---	25.34	27.82	6.5	34	33.86	21.02	17.86

*^a^* Detection of compounds by GC–MS. The results are expressed as the relative abundance based on the total area of the chromatograph. *^b^* Detection of compounds detected by HPLC by chemical standards and listed in order of chromatographic elution. *^c^* Essential oil. *^d^* Hydrosol obtained from the hydrodistillation of the EO. *^e^* Ethanol extract. *^f^* Ethyl acetate extract. *^g^* Polar subfraction of the ethanol extract. *^h^* Polar subfraction of the ethyl acetate extract. *^i^* Non-polar subfraction of the ethanol extract. *^j^* Non-polar subfraction of the ethyl acetate extract.

**Table 2:** Total phenols quantification and antioxidant activity of *P. longiflora* Mexican oregano essential oil, hydrosol and extracts from waste solid residues ^a^.

Analysis	EO *^c^*	HS *^d^*	EOH *^e^*	EAc *^f^*	PSOH *^g^*	PSAc *^h^*	NPSOH *^i^*	NPSAc *^j^*
Total phenols *^b^* (mg GAE/g)	27.85 ± 0.15 *^a^*	0.04 ± 0.00 *^f^*	11.81 ± 0.08 *^b^*	6.13 ± 0.06 *^c^*	0.33 ± 0.00 *^g^*	0.06 ± 0.00 *^h^*	3.77 ± 0.05 *^e^*	4.66 ± 0.09 *^d^*
Iron reduction *^b^* (mg AAE/g)	136.05 ± 0.05 *^a^*	14.16 ± 0.01 *^e^*	26.10 ± 1.45 *^b^*	12.29 ± 1.26 *^c^*	2.84 ± 0.55 *^d^*	0.32 ± 0.08 *^e^*	16.95 ± 2.34 *^c^*	21.18 ± 2.33 *^b^*
IC_50_ *^b^* (μg/mL DPPH)	83.70 ± 4.12 *^c^*	225.00 ± 9.43 *^b^*	151.90 ± 6.65 *^b^*	208.60 ± 12.25 *^b^*	ND	ND	447.20 ± 7.17 *^a^*	ND

*^a^* Different letters within the same line are statistically different (*p* > 0.05). *^b^* Mean ± standard deviation from three independent measurements. *^c^* Essential oil. *^d^* Hydrosol obtained by the hydrodistillation of EO. *^e^* Ethanol extract. *^f^* Ethyl acetate extract. *^g^* Polar subfraction of the ethanol extract. *^h^* Polar subfraction of the ethyl acetate extract. *^i^* Non-polar subfraction of the ethanol extract. *^j^* Non-polar subfraction of the ethyl acetate extract.

**Table 3:** Antimicrobial activity of *P. longiflora* Mexican oregano essential oil (EO), hydrosol, and extracts from waste solid residues, and the minimal inhibitory concentration and minimal cactericidal concentration (mg/L).

Microorganisms	EO *^a^*	HS *^b^*	EOH *^c^*	EAc *^d^*	PSOH *^e^*	PSAc *^f^*	NPSOH *^g^*	NPSAc *^h^*
	Minimal Inhibitory Concentration (mg/L)							
*S. aureus*	250	1000	1000	750	750	500	ND *^j^*	ND
*B. cereus*	250	3000	750	500	750	500	ND	ND
*L. monocytogenes*	500	NI	NI	NI	NI	750	ND	ND
*S.* Typhimurium	500	NI	NI	NI	NI	750	ND	ND
*E. coli* O157:H7	500	NI	1000	750	1000	750	ND	ND
	Minimal Bactericidal Concentration (mg/L)							
*S. aureus*	500	1000	1000	1000	1000	750	1000	750
*B. cereus*	500	3000	1000	750	1000	750	750	1000
*L. monocytogenes*	500	NI	NI	NI	NI	1000	NI	1000
*S.* Typhimurium	750	NI	NI	NI	NI	1000	NI	1000
*E. coli* O157:H7	750	NI	1000	1000	NI	1000	1000	1000

*^a^* Essential oil. *^b^* Hydrosol obtained by the hydrodistillation of EO. The highest concentration tested was 3000 mg/L. *^c^* Ethanol extract. *^d^* Ethyl acetate extract. *^e^* Polar subfraction of the ethanol extract. *^f^* Polar subfraction of the ethyl acetate extract, *^g^* Non-polar subfraction of the ethanol extract. *^h^* Non-polar subfraction of the ethyl acetate extract. *^i^* No inhibition observed at the highest concentration tested. Except for hydrosol, the highest concentration tested was 1000 mg/L. *^j^* Not determined. These are the results of three independent measurements.
